# Survival prediction in glioblastoma: 10-year follow-up from the Dutch Neurosurgery Quality Registry

**DOI:** 10.1007/s11060-025-05080-3

**Published:** 2025-05-23

**Authors:** Jeroen T. J. M. van Dijck, Hilko Ardon, Rutger K. Balvers, Eelke M. Bos, Lisette Bosscher, H. Bart Brouwers, Vincent K. Y. Ho, Koos Hovinga, Lesley Kwee, Mark ter Laan, Rob J. A. Nabuurs, Pierre A. J. T. Robe, Sarita van Geest, Olivier van der Veer, Niels Verburg, Michiel Wagemakers, Philip C. de Witt Hamer, Mar Rodriguez Girondo, Rishi D. S. Nandoe Tewarie, the Quality Registry Neuro Surgery glioblastoma working group from the Dutch Society of Neurosurgery

**Affiliations:** 1 Department of Neurosurgery, Haaglanden Medical Centre, The Hague, The Netherlands; 2 Department of Neurosurgery, Leiden University Medical Center, Leiden, The Netherlands; 3 Department of Neurosurgery, Elisabeth-Tweesteden Hospital, Tilburg, The Netherlands; 4 Department of Neurosurgery, Erasmus MC Cancer Institute, Erasmus University Medical Centre, Rotterdam, The Netherlands; 5 Department of Neurosurgery, Isala, Zwolle, The Netherlands; 6 Department of Research & Development, Netherlands Comprehensive Cancer Organisation (IKNL), Utrecht, The Netherlands; 7 Department of Neurosurgery, Maastricht University Medical Centre, Maastricht, The Netherlands; 8 Department of Neurosurgery, Northwest Clinics, Alkmaar, The Netherlands; 9 Department of Neurosurgery, Radboud University Medical Centre, Nijmegen, The Netherlands; 10 Department of Neurosurgery, University Medical Centre Utrecht, Utrecht, The Netherlands; 11 Department of Neurosurgery, Martini Hospital, Groningen, The Netherlands; 12 Department of Neurosurgery, Medical Spectrum Twente, Enschede, The Netherlands; 13 Department of Neurosurgery, Amsterdam University Medical Centre, Amsterdam, The Netherlands; 14 Department of Neurosurgery, University Medical Centre Groningen, Groningen, The Netherlands; 15 Section of Medical Statistics, Department of Biomedical Data Sciences, Leiden University Medical Center, Leiden, The Netherlands

**Keywords:** Glioblastoma, Patient outcome, Survival, Prognostic model

## Abstract

**Purpose:**

Glioblastoma is the most common and treatment-resistant primary malignant brain tumor, with high morbidity and mortality despite standard treatment protocols. This study aims to evaluate survival and prognostic factors, and introduce two pragmatic prognostic models to inform individualized, patient-centered decision-making, using a large Dutch registry.

**Methods:**

We analyzed a prospective cohort of 7621 patients (2012–2022) in 12 Dutch centers via the Dutch Quality Registry Neurosurgery. Univariate analysis of prognostic factors, Kaplan–Meier survival curves, and funnel plots comparing center performance (30-day and 2-year mortality) were conducted. Two prognostic models using multivariate Cox regression were developed.

**Results:**

Glioblastoma incidence was 3.9/100.000 in The Netherlands. Overall, 30-day mortality was 5.1% and 2-year survival 17.8%. Overall median survival was 10.4 months, with 4.6 months after biopsy and 12.9 months post-resection. Poorer survival correlated with older age, higher ASA classification, lower Karnofsky Performance Status, biopsy over resection (HR 0.49, 95% CI 0.47–0.52), and postoperative complications (HR 1.57 95% CI 1.39–1.79). MGMT promotor methylation (HR 0.58, 95% CI 0.53–0.63) and adjuvant treatment were linked to lower mortality. Treatment variation and outcomes were within expected ranges; surgical volume did not affect survival. The prognostic models had C-indices of 0.704 (6-month) and 0.721 (2-year).

**Conclusion:**

Surgical resection and adjuvant therapy improved survival, but prognosis remained poor. Age, premorbid condition, treatment and molecular markers influenced survival. Center variations were within expected range, and higher surgical volume did not improve outcomes. The developed prognostic models could potentially inform clinicians, pending external validation.

**Supplementary Information:**

The online version contains supplementary material available at 10.1007/s11060-025-05080-3.

## Introduction

Glioblastoma is the most common treatment-resistant primary brain tumor, affecting 1000 Dutch patients annually [1, 2]. It has a 2 year survival of 18% (CI 14–22%) and a 5 year survival of 4% (CI 2–5%) [3]. Standard treatment includes maximal safe surgical resection followed by adjuvant radiotherapy and chemotherapy (Temozolomide) [4]. This Stupp protocol improved 2-year survival from 10.5 to 26.5% [5, 6]. Patients ineligible for resection due to tumor inoperability or poor health undergo biopsy, followed by adjuvant therapy or best supportive care, with significantly worse survival.

Surgeons face complex decisions, weighing the goal of maximal resection benefits against neurological risks that may reduce survival and quality of life [7, 8]. Established prognostic factors include age, Karnofsky Performance Status (KPS), and radiological features (tumor volume, multifocality, eloquence, and mass effect) [9, 10]. However, integrating these factors for treatment guidance remains unclear. For instance, elderly patients may maintain excellent functional status [11]. Moreover, tumor size, location, and associated mass effect or edema can reduce KPS, which may then improve after surgery. Additionally, decisions are often shaped by surgeon experience and multidisciplinary tumor board discussions [7]. This combination of prognostic uncertainty and variability in patients’ and surgeons’ preferences results in highly differing treatment approaches. Consequently, similar patients may receive markedly different care depending on the surgeon or hospital, potentially affecting patient outcomes [12–14].

Most existing prognostic tools for glioblastoma are derived from limited or non-representative datasets and often exclude key molecular markers such as IDH and MGMT, which are now central to diagnosis and prognosis. By leveraging a large, nationwide cohort from all 12 Dutch neurosurgical centers (2012–2022), this study addresses key limitations in glioblastoma research, compares overall survival between centers, re-evaluates prognostic factors, and developed and internally validated two pragmatic prognostic models.

## Methods

### Data source and patients

This nationwide prospective cohort study used Dutch Quality Registry for Neuro Surgery (QRNS) data from 12 neurosurgery centers [12–15]. All patients (≥ 18 years) undergoing first-time glioblastoma surgery (2012–2022) were included. There were no other criteria. Diagnosis followed applicable WHO classification criteria [16–18]. Data included patient demographics [19], functional status (pre/postoperative KPS [20]), surgical details (biopsy/resection, complications), and histopathological markers (MGMT, IDH, TERT, EGFR). Biomarker collection started in 2017. Hospital characteristics include surgical case volume. IDH-mutant tumors were included (n = 158, 2.1%), though status was unavailable for patients < 2017) and because this group is part of the general glioblastoma population presenting in specialized centers. Treatment decisions were multidisciplinary, and biopsy was defined as a procedure solely for diagnostic tissue extraction, including needle and open biopsies. Mortality data was updated in March 2024.

### Statistical analysis

Time to death from the day of surgery was the primary outcome of interest and all analyses were based on complete cases regarding information on covariates. Kaplan–Meier curves and Cox regression models assessed survival patterns by patient characteristics, treatment groups, and center-specific differences. The proportional hazards (PH) assumption in the Cox models was tested using Schoenfeld residuals. Survival analysis used the date of surgery as starting point and time of death as the endpoint with censoring at the last date of follow-up. Center performance (30-day and 2-year mortality) was analyzed using funnel plots [21]. These plots compared observed and expected mortality rates, while adjusting for patient characteristics (age, sex, KPS, ASA) using logistic regression. The x-axis shows center’s sample size, with 95% and 99% control limits forming the funnel shape. Centers outside these limits were identified as outliers. Small-volume centers (< 50 surgeries per year on average) were assessed for potential performance differences, as there was a hypothesis that they may perform worse. Incidence of glioblastoma was calculated for 2022 using data from the Dutch Central Bureau of Statistics [22].

Prognostic model 1, based on 6,860 patients with complete information, is designed to inform in preoperative decision-making using age, sex, preoperative KPS, ASA classification, and surgery type. Model 2 was based on 2,126 complete cases. This number is smaller because biomarker data was only collected from patients starting in 2017. This model incorporates postoperative factors (complications, postoperative KPS, MGMT, and IDH). Both models were developed using a Cox proportional hazards model.

In all fitted regression models (logistic for studying center differences and Cox for prognostic modeling), KPS was modeled as a continuous variable, assuming a linear effect. The ASA score was treated as a categorical variable, with categories IV (n = 73) and V (n = 24) merged with ASA-III subgroup. For analysis, Clavien–Dindo grades 0–II were grouped as “no complications,” reflecting minor events unlikely to affect long-term outcomes. Higher-grade complications (III–V) were not modeled separately due to their low frequency (Supplementary Table 2).

Both models underwent internal–external validation using leave-one-center-out cross-validation, where each center is excluded once to validate a model trained on the others. Model performance was evaluated based on discrimination and calibration metrics. The discriminatory power of the models was assessed using the C-index, which ranges from 0.5 (no discrimination) to 1.0 (perfect discrimination). The C-index was calculated using a cross-validation procedure, in which each study was sequentially omitted to validate model performance. Calibration was evaluated using calibrations plots for 1-year mortality predictions, with bootstrapping employed to account for optimism bias.

Model predictions can be accessed via an Excel tool (Supplementary File 1) and an online app: www.glioblastomaprognosis.com

### Approvals

Patient consent was not required under Dutch regulations, as anonymized data was collected for quality evaluation [23–25]. Ethical approval (N24.040) was obtained, and data was de-identified by a trusted third party.

## Results

### Patient characteristics and patient outcome

From 2012 to 2022, 7799 patients with a first glioblastoma diagnosis were registered in the Dutch Quality Registry Neurosurgery (QRNS). After excluding 116 cases with missing surgical data, 7683 remained; 62 lacked mortality data, leaving 7621 for analysis. At last follow-up, 587 patients were alive (7.7% right-censoring). In 2022, with 696 entries, the minimum national incidence was 3.9 per 100,000.

Table 1 summarizes patient and clinical characteristics across centers. The mean age at presentation was 62 years, 61% were male, 82.1% had a preoperative KPS ≥ 70, and 93.9% had ASA classification 1–3.

**Table 1:** Preoperative patient characteristics and center distribution

		Total	A	B	C	D	E	F	G	H	I	J	K	L
n		7621	942	1096	458	1051	422	818	570	209	295	511	288	961
Age mean (SD)		61.7 (12.4)	59.4 (13.1)	63.4 (12.5)	60.0 (14.6)	62.4 (12.7)	61.7 (12.2)	60.8 (11.9)	60.9 (12.3)	62.6 (11.2)	62.6 (11.1)	62.1 (12.1)	62.3 (11.4)	62.4 (10.9)
Sex (%)	Male	4628 (60.7)	554 (58.8)	668 (60.9)	249 (54.4)	668 (63.6)	262 (62.1)	516 (63.1)	350 (61.4)	120 (57.4)	171 (58.0)	327 (64.0)	175 (60.8)	568 (59.1)
	Female	2766 (36.3)	329 (34.9)	427 (39.0)	182 (39.7)	380 (36.2)	157 (37.2)	292 (35.7)	197 (34.6)	70 (33.5)	115 (39.0)	183 (35.8)	113 (39.2)	321 (33.4)
	Missing	227 (3.0)	59 (6.3)	1 (0.1)	27 (5.9)	3 (0.3)	3 (0.7)	10 (1.2)	23 (4.0)	19 (9.1)	9 (3.1)	1 (0.2)	0 (0.0)	72 (7.5)
KPS (%) Preoperative	KPS ≤ 40	147 (2.0)	9 (0.9)	13 (1.2)	7 (1.5)	29 (2.8)	5 (1.2)	18 (2.2)	17 (3.0)	12 (5.7)	5 (1.7)	12 (2.4)	13 (4.4)	7 (0.7)
	KPS50	344 (4.5)	50 (5.3)	36 (3.3)	5 (1.1)	53 (5.0)	29 (6.9)	24 (2.9)	57 (10.0)	15 (7.2)	14 (4.7)	24 (4.7)	18 (6.2)	19 (2.0)
	KPS60	666 (8.7)	97 (10.3)	90 (8.2)	14 (3.1)	126 (12.0)	25 (5.9)	74 (9.0)	83 (14.6)	15 (7.2)	16 (5.4)	72 (14.1)	32 (11.1)	22 (2.3)
	KPS70	1116 (14.6)	148 (15.7)	156 (14.2)	85 (18.6)	248 (23.6)	68 (16.1)	159 (19.4)	68 (11.9)	16 (7.7)	31 (10.5)	39 (7.6)	38 (13.2)	60 (6.2)
	KPS80	1874 (24.6)	220 (23.4)	336 (30.7)	82 (17.9)	282 (26.8)	111 (26.3)	201 (24.6)	123 (21.6)	48 (23.0)	68 (23.1)	130 (25.4)	57 (19.8)	216 (22.5)
	KPS90	2495 (32.7)	259 (27.5)	378 (34.5)	128 (27.9)	261 (24.8)	140 (33.2)	208 (25.4)	163 (28.6)	79 (37.8)	114 (38.6)	195 (38.2)	90 (31.2)	480 (49.9)
	KPS100	774 (10.2)	144 (15.3)	84 (7.7)	75 (16.4)	48 (4.6)	43 (10.2)	112 (13.7)	53 (9.3)	10 (4.8)	46 (15.6)	17 (3.3)	39 (13.5)	103 (10.7)
	Missing	205 (2.7)	15 (1.6)	3 (0.3)	62 (13.5)	4 (0.4)	1 (0.2)	22 (2.7)	6 (1.1)	14 (6.7)	1 (0.3)	22 (4.3)	1 (0.3)	54 (5.6)
ASA classification (%)	ASA I	1306 (17.1)	194 (20.6)	94 (8.6)	71 (15.5)	166 (15.8)	53 (12.6)	178 (21.8)	87 (15.3)	59 (28.2)	50 (16.9)	92 (18.0)	14 (4.9)	248 (25.8)
	ASA II	4401 (57.7)	541 (57.4)	715 (65.2)	257 (56.1)	640 (60.9)	234 (55.5)	372 (45.5)	353 (61.9)	95 (45.5)	151 (51.2)	298 (58.3)	204 (70.8)	541 (56.3)
	ASA III	1455 (19.1)	174 (18.5)	214 (19.5)	84 (18.3)	230 (21.9)	101 (23.9)	184 (22.5)	113 (19.8)	41 (19.6)	40 (13.6)	82 (16.0)	60 (20.8)	132 (13.7)
	ASA IV	73 (1.0)	2 (0.2)	8 (0.7)	3 (0.7)	13 (1.2)	5 (1.2)	10 (1.2)	7 (1.2)	2 (1.0)	4 (1.4)	5 (1.0)	7 (2.4)	7 (0.7)
	ASA V	24 (0.3)	6 (0.6)	4 (0.4)	0 (0.0)	0 (0.0)	4 (0.9)	0 (0.0)	0 (0.0)	4 (1.9)	2 (0.7)	0 (0.0)	1 (0.3)	3 (0.3)
	Missing	362 (4.8)	25 (2.7)	61 (5.6)	43 (9.4)	2 (0.2)	25 (5.9)	74 (9.0)	10 (1.8)	8 (3.8)	48 (16.3)	34 (6.7)	2 (0.7)	30 (3.1)
Surgery (%)	Biopsy	2420 (31.8)	247 (26.2)	312 (28.5)	255 (55.7)	300 (28.5)	156 (37.0)	200 (24.4)	158 (27.7)	64 (30.6)	122 (41.4)	117 (22.9)	78 (27.1)	411 (42.8)
	Resection	5201 (68.2)	695 (73.8)	784 (71.5)	203 (44.3)	751 (71.5)	266 (63.0)	618 (75.6)	412 (72.3)	145 (69.4)	173 (58.6)	394 (77.1)	210 (72.9)	550 (57.2)

Follow-up (median 2 years) ranged from under 1 month to 10 years. Median overall survival was 10.4 months (95%CI 10.1–10.7), varying between 8.2 and 11.8 months across centers. The 30-day mortality rate was 5.1% (range 4.0–7.6%), and 2-year survival was 17.8% (range 13.4–21.4%). In the biopsy group, median survival was 4.6 months with 9.9% 30-day mortality. In the resection group 12.9 months with 2.9% 30-day mortality. Two-year survival was 8.4% and 22.2% respectively (Table 2).

**Table 2:** Postoperative patient and center characteristics

		Total	A	B	C	D	E	F	G	H	I	J	K	L
n		7621	942	1096	458	1051	422	818	570	209	295	511	288	961
Complications (%)	No	6622 (86.9)	876 (93.0)	995 (90.8)	82 (17.9)	995 (94.7)	383 (90.8)	727 (88.9)	501 (87.9)	201 (96.2)	242 (82.0)	413 (80.8)	281 (97.6)	926 (96.4)
	Yes	261 (3.4)	40 (4.2)	42 (3.8)	5 (1.1)	53 (5.0)	5 (1.2)	26 (3.2)	22 (3.9)	6 (2.9)	7 (2.4)	15 (2.9)	6 (2.1)	34 (3.5)
	Missing	738 (9.7)	26 (2.8)	59 (5.4)	371 (81.0)	3 (0.3)	34 (8.1)	65 (7.9)	47 (8.2)	2 (1.0)	46 (15.6)	83 (16.2)	1 (0.3)	1 (0.1)
KPS (%) postoperative	KPS ≤ 40	128 (1.6)	10 (1.0)	16 (1.5)	6 (1.4)	23 (2.2)	5 (1.2)	17 (2.0)	12 (2.2)	4 (2.0)	2 (0.6)	10 (2.0)	13 (4.4)	10 (1.0)
	KPS50	445 (5.8)	48 (5.1)	76 (6.9)	5 (1.1)	57 (5.4)	41 (9.7)	27 (3.3)	78 (13.7)	15 (7.2)	12 (4.1)	42 (8.2)	25 (8.7)	19 (2.0)
	KPS60	650 (8.5)	82 (8.7)	100 (9.1)	18 (3.9)	128 (12.2)	39 (9.2)	72 (8.8)	72 (12.6)	18 (8.6)	11 (3.7)	68 (13.3)	27 (9.4)	15 (1.6)
	KPS70	997 (13.1)	125 (13.3)	143 (13.0)	47 (10.3)	312 (29.7)	73 (17.3)	83 (10.1)	73 (12.8)	10 (4.8)	19 (6.4)	22 (4.3)	41 (14.2)	49 (5.1)
	KPS80	1510 (19.8)	182 (19.3)	291 (26.6)	42 (9.2)	296 (28.2)	122 (28.9)	105 (12.8)	120 (21.1)	41 (19.6)	42 (14.2)	41 (8.0)	48 (16.7)	180 (18.7)
	KPS90	2027 (26.6)	245 (26.0)	368 (33.6)	71 (15.5)	201 (19.1)	111 (26.3)	130 (15.9)	150 (26.3)	89 (42.6)	70 (23.7)	175 (34.2)	90 (31.2)	327 (34.0)
	KPS100	475 (6.2)	77 (8.2)	70 (6.4)	49 (10.7)	16 (1.5)	12 (2.8)	40 (4.9)	50 (8.8)	4 (1.9)	15 (5.1)	45 (8.8)	39 (13.5)	58 (6.0)
	Missing	1389 (18.2)	173 (18.4)	32 (2.9)	220 (48.0)	18 (1.7)	19 (4.5)	344 (42.1)	15 (2.6)	28 (13.4)	124 (42.0)	108 (21.1)	5 (1.7)	303 (31.5)
Treatment (%)	Chemo radio therapy	4325 (56.8)	609 (64.6)	639 (58.3)	146 (31.9)	586 (55.8)	240 (56.9)	380 (46.5)	325 (57.0)	113 (54.1)	160 (54.2)	281 (55.0)	209 (72.6)	637 (66.3)
	Radio therapy	899 (11.8)	70 (7.4)	157 (14.3)	62 (13.5)	164 (15.6)	52 (12.3)	67 (8.2)	113 (19.8)	35 (16.7)	43 (14.6)	30 (5.9)	5 (1.7)	101 (10.5)
	Chemo therapy	414 (5.4)	96 (10.2)	38 (3.5)	54 (11.8)	75 (7.1)	15 (3.6)	46 (5.6)	9 (1.6)	3 (1.4)	13 (4.4)	28 (5.5)	16 (5.6)	21 (2.2)
	No treatment	1605 (21.1)	162 (17.2)	254 (23.2)	70 (15.3)	226 (21.5)	112 (26.5)	163 (19.9)	116 (20.4)	57 (27.3)	77 (26.1)	108 (21.1)	58 (20.1)	202 (21.0)
	Missing	378 (5.0)	5 (0.5)	8 (0.7)	126 (27.5)	0 (0.0)	3 (0.7)	162 (19.8)	7 (1.2)	1 (0.5)	2 (0.7)	64 (12.5)	0 (0.0)	0 (0.0)

Biomarkers		Total	A	B	C	D	E	F	G	H	I	J	K	L
n		4421	497	691	280	581	266	449	336	114	173	333	174	527
MGMT (%)	No Methylated (−)	1761 (39.8)	170 (34.2)	461 (66.7)	129 (46.1)	245 (42.2)	129 (48.5)	157 (35.0)	118 (35.1)	51 (44.7)	36 (20.8)	116 (34.8)	100 (57.5)	49 (9.3)
	Methylated (+)	1213 (27.4)	124 (24.9)	198 (28.7)	126 (45.0)	145 (25.0)	121 (45.5)	141 (31.4)	122 (36.3)	45 (39.5)	18 (10.4)	75 (22.5)	67 (38.5)	31 (5.9)
	Missing	1447 (32.7)	203 (40.8)	32 (4.6)	25 (8.9)	191 (32.9)	16 (6.0)	151 (33.6)	96 (28.6)	18 (15.8)	119 (68.8)	142 (42.6)	7 (4.0)	447 (84.8)
IDH (%)	Wild type (−)	3674 (83.1)	408 (82.1)	642 (92.9)	231 (82.5)	523 (90.0)	255 (95.9)	261 (58.1)	295 (87.8)	98 (86.0)	163 (94.2)	291 (87.4)	158 (90.8)	349 (66.2)
	Mutant (+)	158 (3.6)	19 (3.8)	16 (2.3)	26 (9.3)	22 (3.8)	8 (3.0)	14 (3.1)	8 (2.4)	3 (2.6)	6 (3.5)	8 (2.4)	8 (4.6)	20 (3.8)
	Missing	589 (13.3)	70 (14.1)	33 (4.8)	23 (8.2)	36 (6.2)	3 (1.1)	174 (38.8)	33 (9.8)	13 (11.4)	4 (2.3)	34 (10.2)	8 (4.6)	158 (30.0)
TERT (%)	Wild type (−)	237 (5.4)	9 (1.8)	83 (12.0)	6 (2.1)	41 (7.1)	24 (9.0)	22 (4.9)	5 (1.5)	2 (1.8)	7 (4.0)	16 (4.8)	4 (2.3)	18 (3.4)
	Mutant (+)	1187 (26.8)	30 (6.0)	414 (59.9)	37 (13.2)	170 (29.3)	138 (51.9)	132 (29.4)	17 (5.1)	8 (7.0)	54 (31.2)	89 (26.7)	16 (9.2)	82 (15.6)
	Missing	2997 (67.8)	458 (92.2)	194 (28.1)	237 (84.6)	370 (63.7)	104 (39.1)	295 (65.7)	314 (93.5)	104 (91.2)	112 (64.7)	228 (68.5)	154 (88.5)	427 (81.0)
EGFR (%)	No amplified (−)	941 (21.3)	141 (28.4)	283 (41.0)	8 (2.9)	139 (23.9)	139 (52.3)	0 (0.0)	70 (20.8)	22 (19.3)	29 (16.8)	60 (18.0)	10 (5.7)	40 (7.6)
	Amplified (+)	792 (17.9)	103 (20.7)	192 (27.8)	8 (2.9)	74 (12.7)	74 (27.8)	0 (0.0)	157 (46.7)	69 (60.5)	20 (11.6)	50 (15.0)	7 (4.0)	38 (7.2)
	Missing	2688 (60.8)	253 (50.9)	216 (31.3)	264 (94.3)	368 (63.3)	53 (19.9)	449 (100.0)	109 (32.4)	23 (20.2)	124 (71.7)	223 (67.0)	157 (90.2)	449 (85.2)

Outcome	B*	R*	Total	A	B	C	D	E	F	G	H	I	J	K	L
Median survival (m)	4.6 (4.4–5.0)	12.9 (12.6–13.3)	10.4 (10.1–10.7)	11.8 (11.2–13.0)	10.4 (9.7–10.9)	10.5 (9.1–11.9)	10.3 (9.7–11.2)	8.8 (7.6–9.7)	10.2 (9.4–10.9)	9.9 (9.3–11.2)	10.0 (7.9–12.6)	8.2 (7.4–12.3)	10.4 (9.4–11.4)	10.8 (9.8–12.2)	10.7 (9.8–11.8)
30-day mortality %	9.9 (8.7–11.1)	2.9 (2.4–3.3)	5.1 (4.6–5.6)	4.0 (2.8–5.3)	5.1 (3.8–6.4)	7.6 (5.2–10.0)	5.7 (4.3–7.1)	6.2 (3.8–8.4)	4.5 (3.1–5.9)	5.1 (3.3–6.9)	5.7 (2.5–8.8)	4.4 (2.0–6.7)	5.3 (3.3–7.2)	6.2 (3.4–9.0)	4.0 (2.7–5.2)
2 year survival %	8.4 (7.3–9.6)	22.2 (21.1–23.4)	17.8 (16.9–18.7)	19.3 (16.9–22.1)	17.7 (15.5–20.2)	21.4 (17.9–25.5)	18.6 (16.3–21.2)	14.6 (11.6–18.5)	16.8 (14.5–19.6)	17.8 (14.9–21.3)	17.8 (13.2–23.9)	13.4 (10.0–18.0)	17.7 (14.7–21.4)	17.6 (13.7–22.6)	17.5 (15.2–20.1)

Biomarker data was available from 2017 to 2022; missings refer to this period

*B* biopsy, *R* resection

### Patient treatment

A total of 5201 patients (68.2%) underwent surgical resection, with center-specific rates ranging from 44.3 to 77.1%, while 2420 patients (31.8%) received a biopsy, ranging from 22.9 to 55.7% across centers. Combined chemoradiotherapy was initiated in 4325 patients (56.8%, range 31.9–2.6%). Monotherapy was given to 1313 patients (17.2%), of which 899 radiotherapy (11.8%, range 1.7–19.8%) and 414 chemotherapy (5.4%, range 1.4–11.8%). A total of 1605 patients received no adjuvant therapy (21.1%, range 15.3–27.3%) (Table 1).

**Fig. 1:**
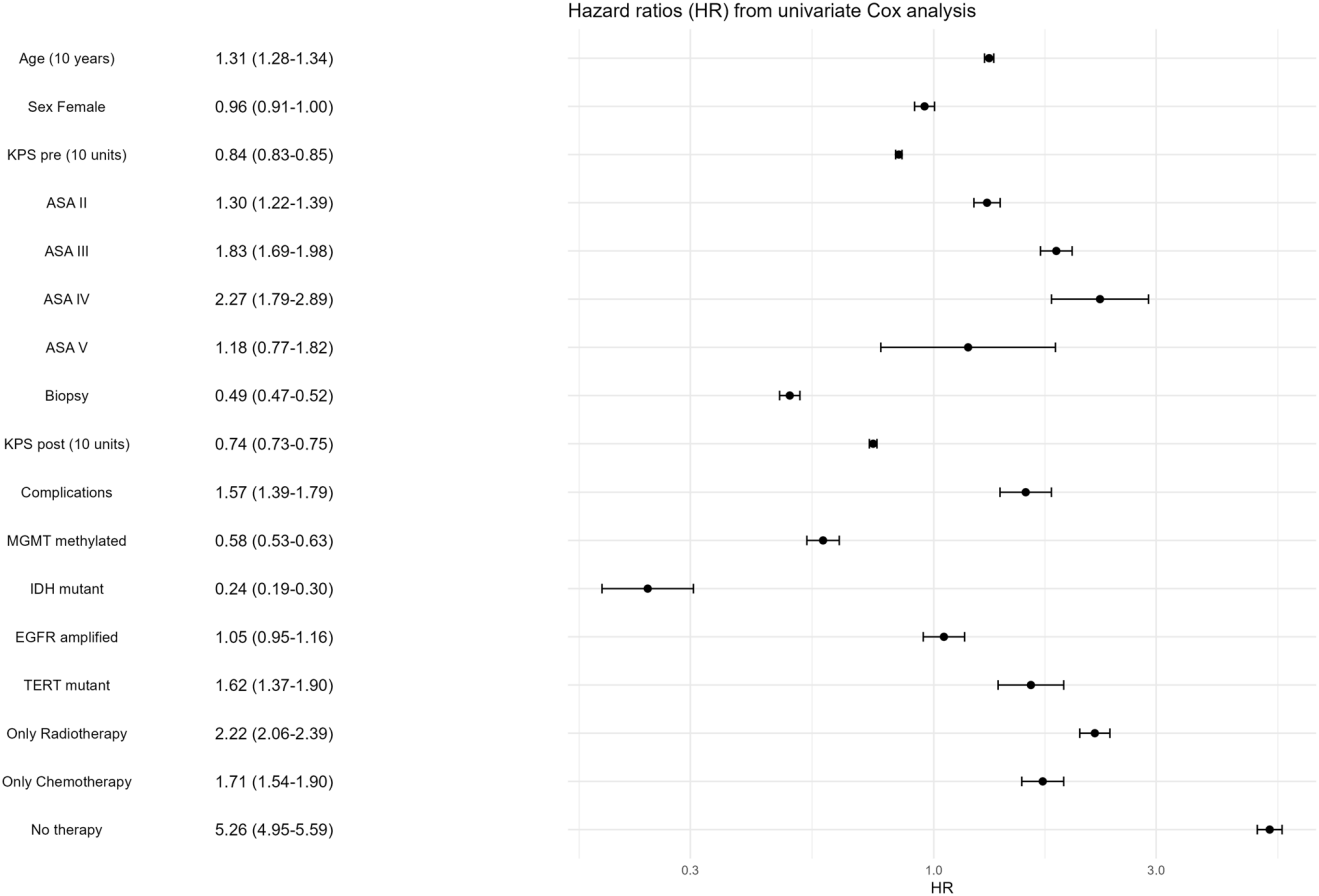
Hazard ratios (HR) for death from univariate Cox analysis

Any type of adjuvant treatment was associated with improved survival in both biopsy and resection subgroups. Patients receiving chemotherapy alone had significantly better survival with MGMT methylation (Supplemental Fig. 1). Combined therapy showed the greatest survival, followed by chemotherapy (HR 1.71, 95%CI 1.54–1.90), radiotherapy (HR 2.22, 95%CI 2.06–2.39), and no adjuvant treatment (HR 5.26, 95%CI 4.95–5.59) (Fig. 1). Median survival of patients not receiving adjuvant therapy after biopsy was 2.5 months (95%CI 2.4–2.7) and after resection 4.0 months (95%CI 3.8–4.3). The Schoenfeld residuals test showed no clear violation of the proportional hazards assumption in any univariate Cox model.

### Between center differences

Beyond treatment variation, patient outcomes differed across the 12 centers. Median overall survival ranged from 8.2 to 11.8 months, 30-day mortality from 4.0 to 7.6%, and 2-year survival from 13.4 to 21.4% (Table 2).

**Fig. 2:**
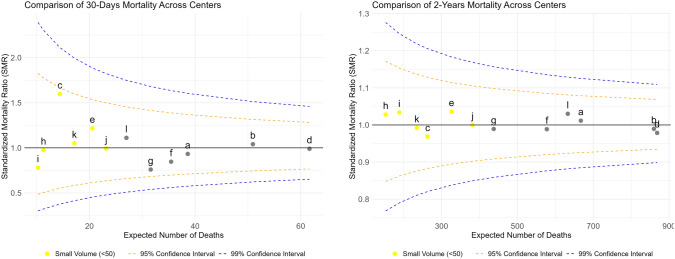
Among center differences in 30-day mortality and 2-year survival. The x-axis represents the center’s sample size, and the funnel shape was created by control limits at the 95% and 99% confidence levels. Yellow squares are small volume centers (< 50 operated cases annually)

After adjusting for risk factors, no center was a significant outlier for 30-day or 2-year mortality. Low-volume hospitals (< 50 cases) also showed no worse outcomes (Fig. 2).

### Prognostic factors

Higher age (per 10 years HR 1.31 95% CI 1.28–1.34), higher ASA classification, lower preoperative performance status (KPS pre) (per 10 units HR 0.84 95% CI 0.83–0.85), biopsy over resection (HR 0.49, 95% CI 0.47–0.52) were associated with a shorter survival. Sex was not associated with survival. In addition, occurrence of postoperative complications (HR 1.57 95% CI 1.39–1.79) and lower postoperative functional status (KPS post) (per 10 units HR 0.74 95% CI 0.73–0.75) were associated with shorter survival (Fig. 1 & Supplemental Fig. 2).

### Molecular markers

In The Netherlands, biomarker registration started from 2017 onwards. IDH marker examination showed 83.1% wildtype tumors, with 3.6% mutant and 13.3% missings. The number of missing IDH markers decreased from 172 cases in 2017 to 41 cases in 2022. Other markers were only tested in a specific population of patients, based on e.g. functional status and age. Between centers, IDH testing ranged from 58.1 to 94.2% and MGMT from 9.3 to 66.7%. Established molecular markers were linked to survival outcomes. MGMT promotor methylation and IDH mutant were both linked to lower mortality rates (HR 0.58, 95% CI 0.53–0.63 and HR 0.24, 95% CI 0.19–0.30, respectively). TERT promotor mutation was associated with a higher mortality (HR 1.62, 95% CI 1.37–1.90), while EGFR status had no significant impact on survival (Fig. 1 and Supplemental Fig. 3).

### Prognostic models

**Fig. 3:**
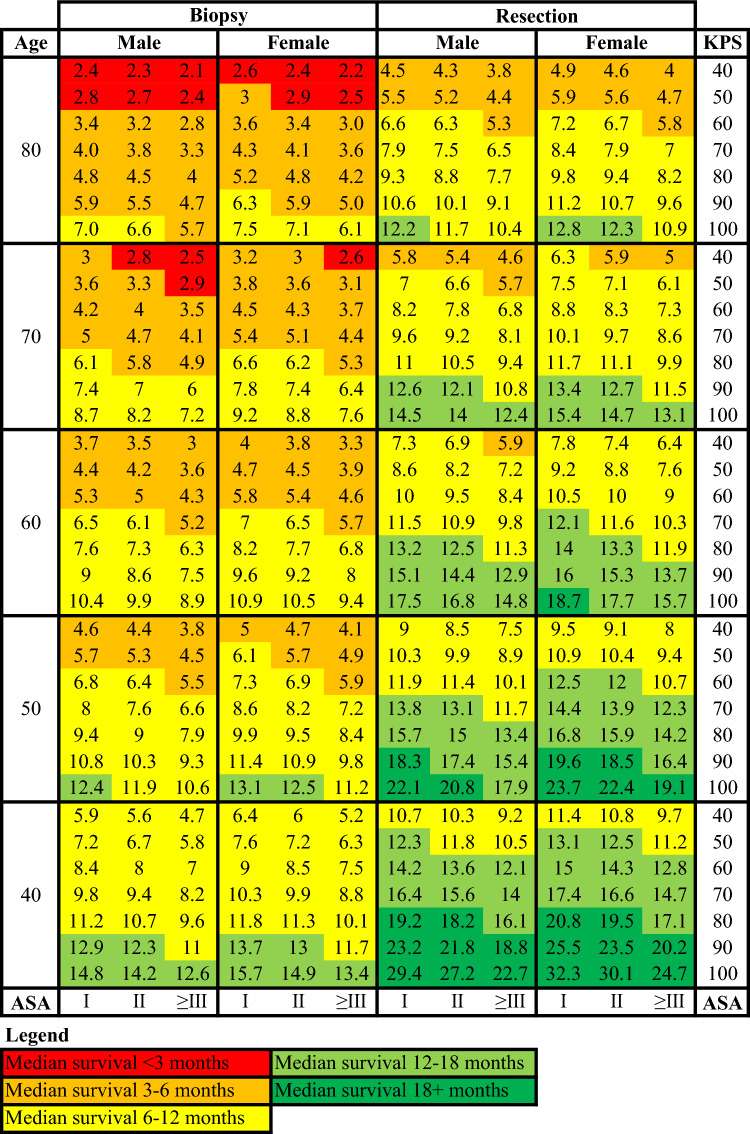
Chart of predicted survival in months according to prognostic model 1 involving type of surgery, preoperative KPS, ASA and age

Prognostic model 1 estimates survival after biopsy and resection. Regression coefficients (Supplemental Table 1) and calibration plots (Supplemental Fig. 4) are provided. A 40-year old male (ASA-I, KPS 40) has a predicted survival of 5.9 months with biopsy vs. 10.7 months with resection. Structured charts for survival estimations can be found in Fig. 3. The model’s C-index is 0.704 at 6 months, declining to 0.680 at 1 year, indicating moderate predictive ability.

Prognostic model 2 predicts survival after incorporating additional postoperative variables. Regression coefficients, calibration plots (Supplemental Fig. 5), and survival predictions (Supplemental Figs. 6–9) are available. For example, a 60-year-old female (ASA-I, postoperative KPS 80, no complications, MGMT-unmethylated, IDH-wildtype) has median survival of 12.2 months post-resection. The model’s C-index is 0.770 at 6 months, 0.736 at 1 year, and 0.715 at 2 years, demonstrating strong discriminatory ability.

Both prognostic models demonstrate reasonable calibration, with predicted and observed patient proportions aligning across different risk levels. However, miscalibration is observed in the lower-risk groups, where the predictions tend to be overly optimistic.

## Discussion

This study found that 2-year survival after initial glioblastoma surgery remains poor, reflecting the disease’s aggressiveness. Prognostic factors like age, premorbid condition, treatment strategies, and molecular markers (MGMT, IDH, TERT) were associated with survival. Treatment variation across centers did not significantly impact outcomes. Higher surgical case volume (> 50 patients) was not associated with improved survival or lower mortality.

### Patient outcome and treatment variation

The 2-year survival rate was 17.8%, higher than the 13.5% previously reported in Dutch data [12], and comparable to England (11.5%) [26], the U.S. (18.7%) [27], Italy (24.8%) [28], and Belgium (21.3%) after resection [29]. Differences likely reflect case-mix and treatment variations. A recent meta-analysis found 2-year survival increased from 9% (CI 6–12%) before 2005 to 18% (CI 14–22%) after 2005 [3], likely due to advances in surgery, adjuvant therapies (e.g., Stupp protocol), and integration of molecular markers with the revised WHO glioma classification. Still, mortality remains high.

High mortality was associated with several clinical factors, with age being the strongest predictor, consistent with prior literature [10, 12, 26]. This may reflect reduced treatment intensity, lower therapeutic response, increased toxicity, altered tumor biology, and exclusion from clinical trials [6, 11]. Outcomes also varied by treatment strategy, likely affected by confounding by indication. Survival was higher after resection than biopsy. Median survival in the biopsy group was 4.6 months, with 9.9% 30-day mortality. Without adjuvant therapy, median survival dropped to 2.5 months. Chemoradiotherapy significantly improved survival compared to no adjuvant treatment or monotherapy. These findings align with previous studies showing best outcomes with maximal treatment [6, 26, 30, 31].

Consistent with previous studies [10], MGMT promoter methylation was associated with lower mortality, aligning with a pooled HR of 1.66 (95% CI 1.32–2.09) from a recent meta-analysis [32]. IDH mutations also correlated with significantly lower mortality, with an even stronger effect than the pooled HR of 2.37 (95% CI 1.81–3.12) [32]. Including IDH-mutant cases likely improved overall outcomes but is justified by their small number (n = 158, 3.6%), their clinical relevance, and likely even distribution across centers and treatments. Some additional IDH-mutant cases from 2012 to 2016 may have gone undetected, further supporting their inclusion.

The prognostic role of TERT remains debated [33, 34]; though our data suggest a possible link to higher mortality. In contrast to meta-analysis findings, high EGFR expression was not significantly associated with increased mortality in our study [32]. Overall, the role of molecular markers and targeted therapies remains uncertain and is the focus of ongoing research.

Our previous study documented hospital-level differences in 30-day and 2-year survival [12]. In the current analysis, despite the combination of several centers, treatment differences and crude survival variation, no clear outlier centers emerged. One center approached the 95% funnel plot limit for 30-day mortality without clear cause. Higher surgical volume (> 50 cases/year) was not associated with lower mortality, though this threshold—based on Dutch glioblastoma centralization debates—is debatable. A UK study linked surgeon (not center) volume with 30-day mortality [35], while studies from the US and Finland found better outcomes at academic and high-volume centers [36, 37]. However, cross-countries comparisons are limited by differences in case selection and healthcare organization.

### Surgical decision-making

Reducing glioblastoma mortality remains challenging [38], but improving the decision-making process may enhance outcomes for patients and proxies [39–41]. Thorough discussions of risks, benefits, expectations, and goals can reduce anxiety, support emotional well-being, and improve quality of life [39, 40]. Healthcare providers can aid decision-making by offering clear information on prognosis, survival, and quality-of-life outcomes. By developing a clinically relevant, user-friendly prognostic model for survival after sergery, we aim to improve patient involvement and shared decision-making [40].

Prognostic model 1 may support shared decision-making by estimating median survival for resection versus biopsy, aiding choices when both are viable. It may also guide counseling when resection isn’t feasible. Despite a moderate C-index, the model is simple, requiring few inputs. Although the ASA score is not routinely used by neurologists or neurosurgeons, it is straightforward and accessible. This balance of simplicity and utility may promote patient involvement.

Model 2, though requiring more data, remains user-friendly and shows strong discriminatory performance. It may inform decision-making during multidisciplinary meetings or postoperative consultations. While it lacks radiological data and slightly overestimates survival in low-risk patients—particularly younger individuals with favorable profiles, its clinical impact is likely limited [3, 42]. Still, predictions for this group should be interpreted with caution and supplemented by clinical judgment.

Both prognostic models could be improved by incorporating radiologic variables (e.g., tumor volume, location, eloquence, multifocality, mass effect), surgical techniques (e.g., use of intraoperative monitoring), extent of resection, and other yet-unknown factors. In theory, highly accurate models could personalize treatment, but overly complex models may hinder practical use and clinical integration [43]. Greater accuracy however doesn’t always translate into better decisions or outcomes. For example, not every patient will receive surgery and chemoradiotherapy, simply because survival is longer.

Despite several glioblastoma models reporting AUCs of 0.58–0.98 and C-indices of 0.70–0.82, clinical use remains limited [43]. A recent online tool by Senders et al. achieved a C-index of 0.70 and authors suggested that discrimination (0.63–0.77) could be improved with machine and deep learning methods [44]. However, despite growing interest in high-dimensional machine learning models, clinical adoption has lagged. For real-world implementation, future work should focus on standardized variable collection, improved model interpretability, and external validation through multicenter prospective studies.

These prognostic models have not been externally validated and should be used cautiously, as part of a comprehensive, individualized assessment by a qualified clinician [44]. Despite efforts to ensure accuracy, limitations in data completeness and precision remain. External validation in diverse cohorts and impact studies are needed to evaluate their influence on clinical decision-making, patient outcomes, and comparison with standard care [45].

### Strengths and limitations

A major strength of this nationwide, population-based cohort is its large sample size and high-quality, long-term follow-up. Regarding the between center comparison, limitations include potential confounding by indication and unmeasured variables. Although small-volume center may introduce bias, they treated substantial patient numbers annually, and our funnel plot analyses adjusted for key characteristics. Still, some variability remains possible. Missing data on surgical techniques, adjuvant therapies, and treatment adherence restricted analysis, and molecular marker data were only routinely collected from 2017 onward. We recognize that these molecular markers may not always be available in every clinical setting, which may limit the applicability of the proposed predictive models—particularly model 2. In such cases, strategies such as imputation may be considered, although this may come at the cost of predictive accuracy. Nonetheless, given the increasing routine use of MGMT and IDH testing in neuro-oncology, we believe that the proposed prognostic models are becoming increasingly applicable in routine clinical practice.

Also, generalization beyond the Dutch healthcare setting is uncertain. Advances in molecular diagnostics complicate interpretation across heterogeneous cohorts, and glioblastoma incidence may be underestimated due to inclusion of only histologically confirmed cases. Lastly, the lack of functional outcome and quality of life data limits assessment of the clinical relevance of extended survival and the prognostic models await external validation.

## Conclusion

Surgical resection and adjuvant treatment were associated with increased survival in all age groups, but survival remained poor. Survival was influenced by age, premorbid condition, treatment strategies, and molecular markers (MGMT, IDH, TERT). Center-level variation in outcomes and treatment was within expected ranges, and higher surgical volume did not correlate with better survival. The developed prognostic models may have the potential to inform clinicians, pending future external validation.

## Supplementary Information

Below is the link to the electronic supplementary material.

Supplementary file1 (DOCX 1304 KB)

Supplementary file2 (XLSX 175 KB)

## Data Availability

No datasets were generated or analysed during the current study.
