# Supplementary material for: Survival prediction in glioblastoma: 10-year follow-up from the Dutch Neurosurgery Quality Registry
Source: J Neurooncol. 2025 May 23;174(3):753–64. doi: 10.1007/s11060-025-05080-3 (PMC12263716; doi:10.1007/s11060-025-05080-3)

**Supplementary data**

| **Model 1** | |  | **Model 2** | |
| --- | --- | --- | --- | --- |
| Risk factor | Coefficient |  | Risk factor | Coefficient |
| Age* | 0,240 |  | Age* | 0,170 |
| KPS preoperative* | -0,190 |  | KPS postoperative* | -0,300 |
| Sex=Female | -0,075 |  | Sex=Female | -0,084 |
| ASAII | 0,062 |  | ASAII | 0,057 |
| ASA≥III | 0,216 |  | ASA≥III | 0,309 |
| Resection | -0,702 |  | Resection | -0,649 |
|  |  |  | Complications | 0,250 |
|  |  |  | MGMT methylated | -0,638 |
|  |  |  | IDH mutant | -0,708 |

**Supplemental table 1:** Regression coefficients for prognostic models. * = per 10 units.

**Supplemental table 2:** Number of complications

| **Description** | **Number of patients** |
| --- | --- |
| No complications | 5675 |
| Grade I | 570 |
| Grade II | 377 |
| Grade III | 85 |
| Grade IIIa | 7 |
| Grade IIIb | 67 |
| Grade IV | 42 |
| Grade IVa | 23 |
| Grade IVb | 1 |
| Grade V | 36 |

**Supplemental figure 1:**

Kaplan Meier curves from univariate Cox analysis for patient undergoing a biopsy or resection and receiving postoperative chemoradiation, chemotherapy, radiotherapy, or no therapy. The figure also shows survival probabilities for patients undergoing chemotherapy alone with different MGMT status.

**
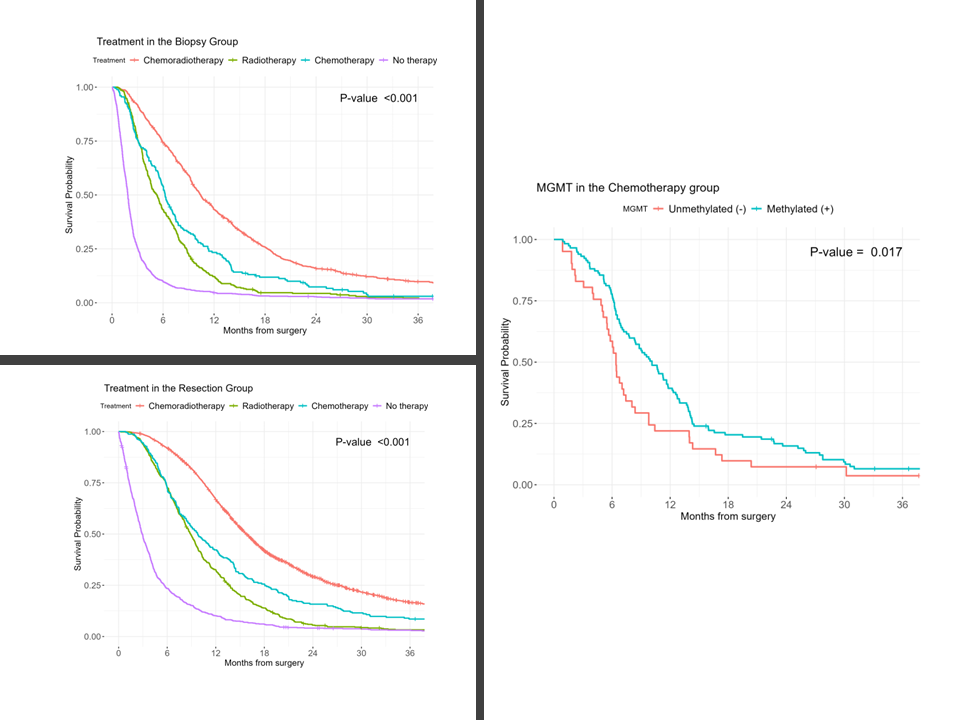
**

**Supplemental figure 2:**

Kaplan Meier curves from univariate Cox analysis of patients stratified on sex, age, type of surgery, complications, and pre- and postoperative KPS.

**
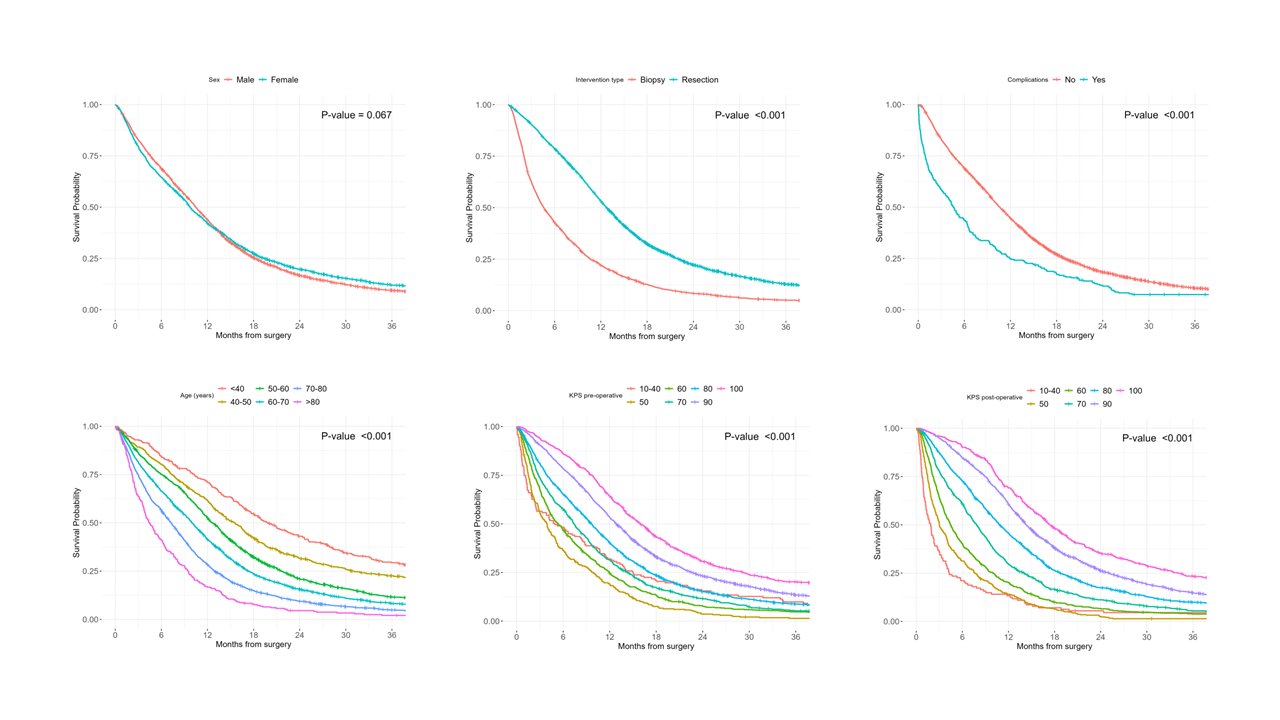
**

**Supplemental figure 3:**

Kaplan Meier curves from univariate Cox analysis of patients stratified on biomarker status: IDH, MGMT, EGFR, and TERT.

**
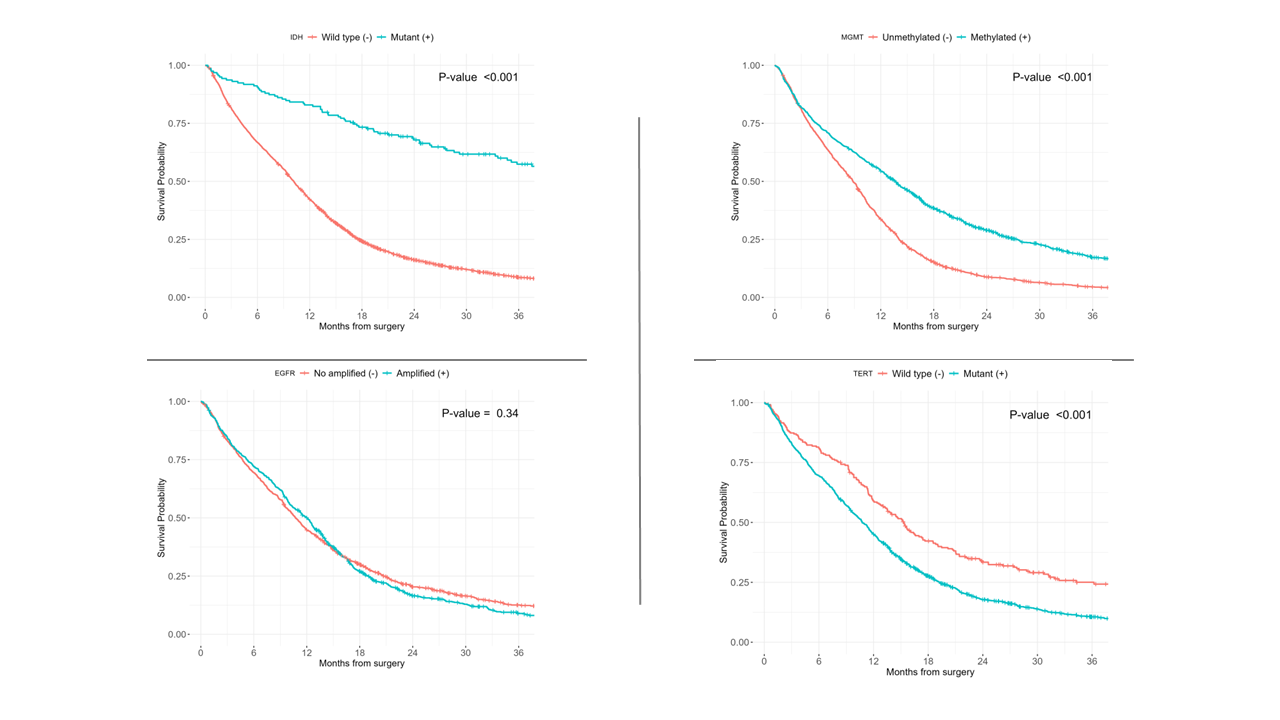
**

**Supplemental figure 4:**

Calibration plot for 1-year survival in model 1.


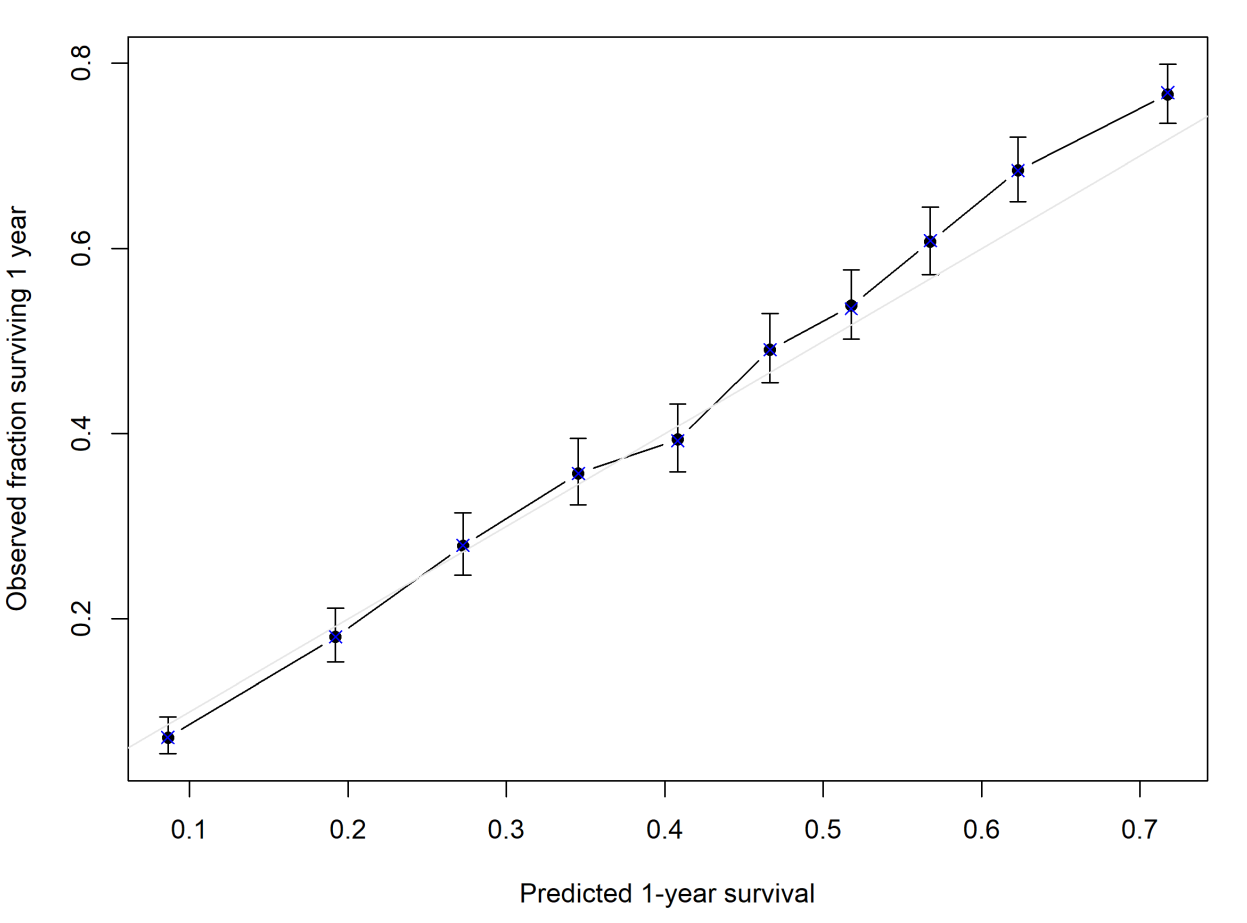


**Supplemental figure 5:**

Calibration plot for 1-year survival in model 2.


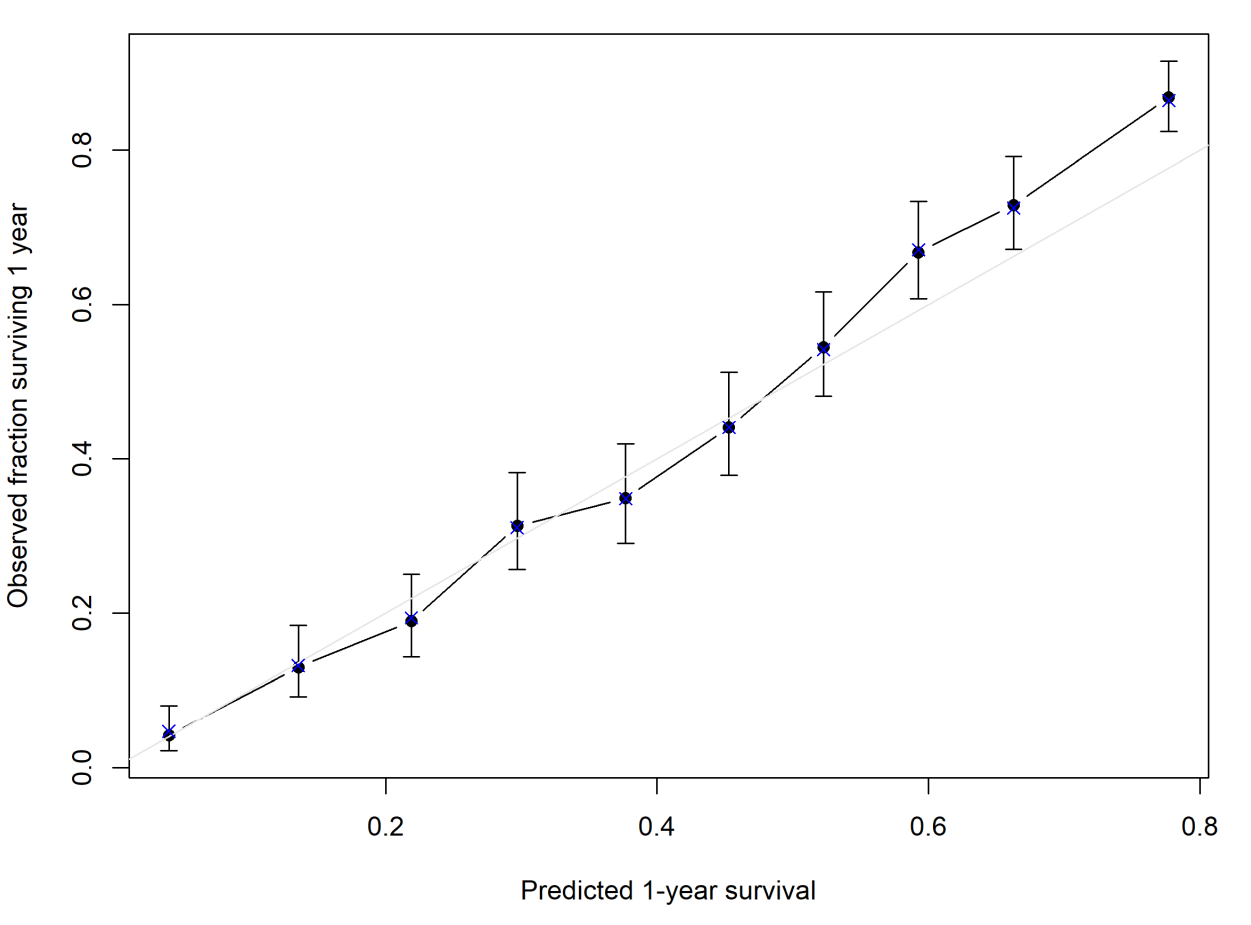


**Supplemental figure 6:** Chart of predicted median survival in months after biopsy in males according to prognostic model 2 involving biomarkers and functional status (postoperative KPS and complications).

**
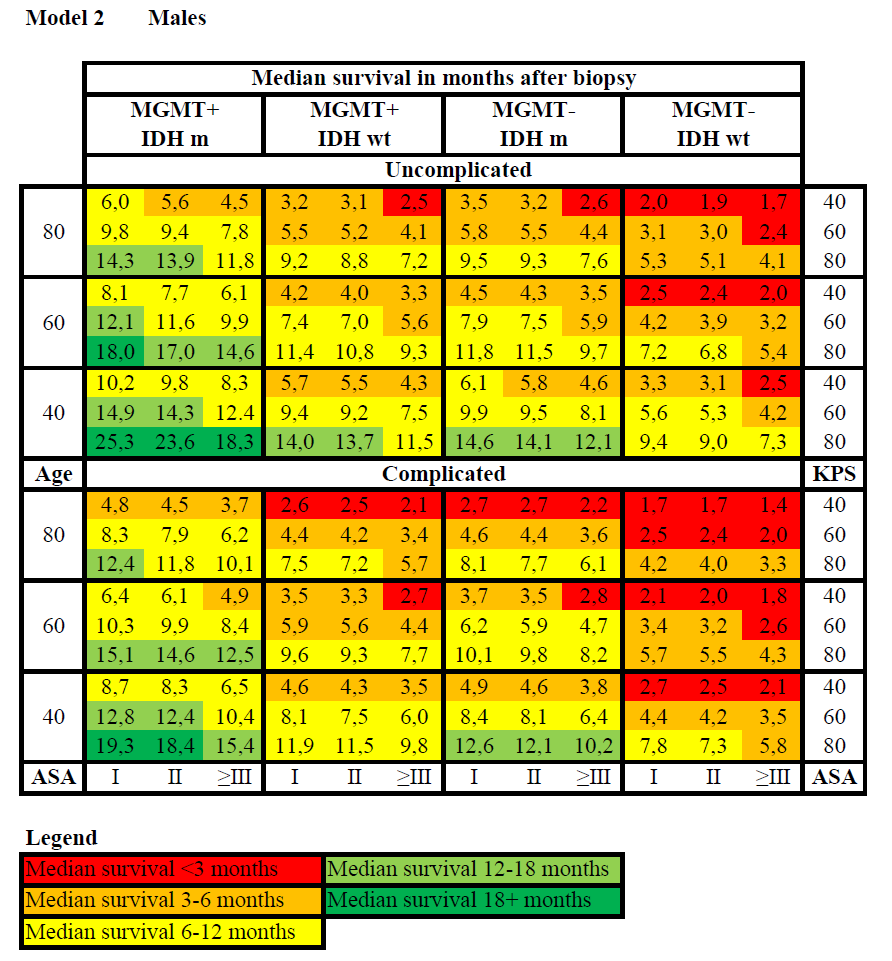
**

**Supplemental figure 7:** Chart of predicted median survival in months after resection in males according to prognostic model 2 involving biomarkers and functional status (postoperative KPS and complications).


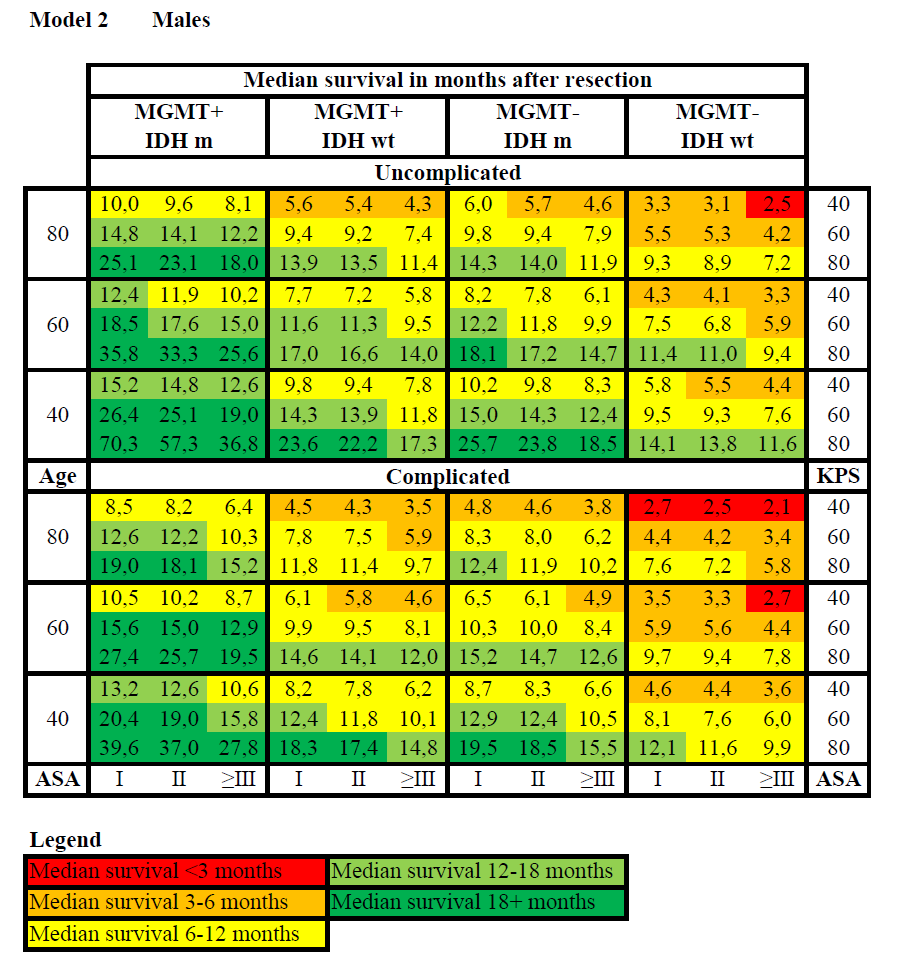


**Supplemental figure 8:** Chart of predicted median survival in months after biopsy in females according to prognostic model 2 involving biomarkers and functional status (postoperative KPS and complications).


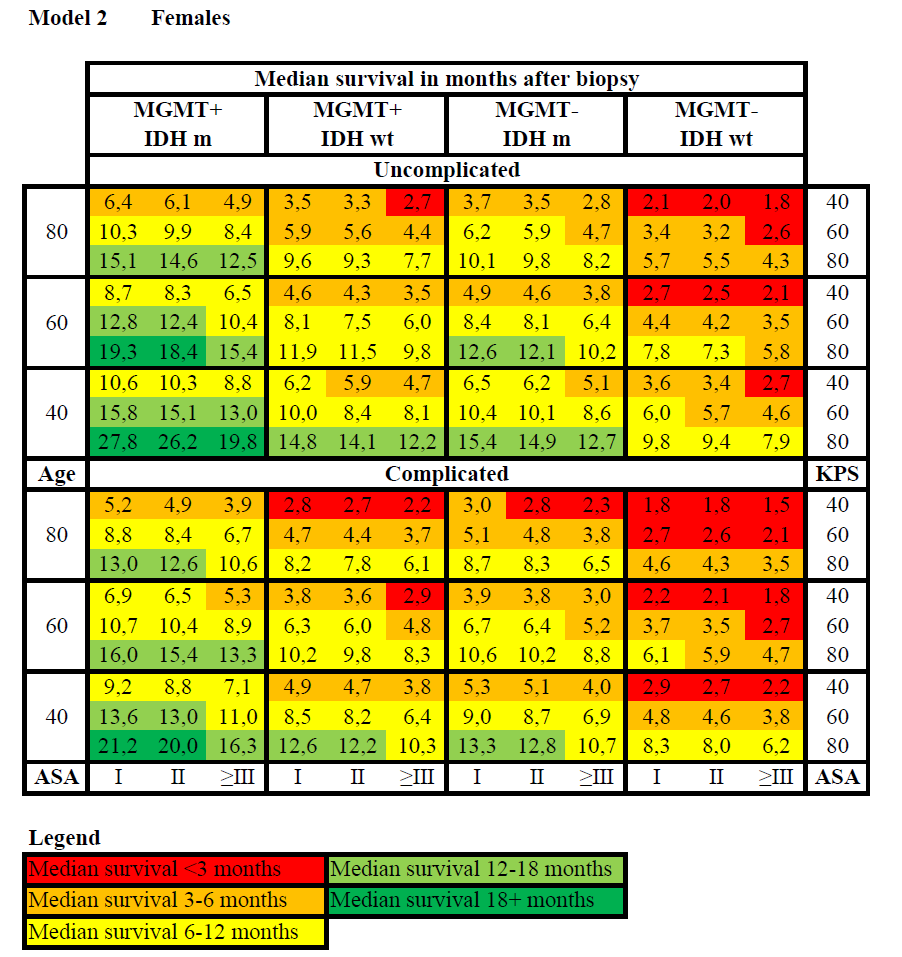


**Supplemental figure 9:** Chart of predicted median survival in months after resection in females according to prognostic model 2 involving biomarkers and functional status (postoperative KPS and complications).


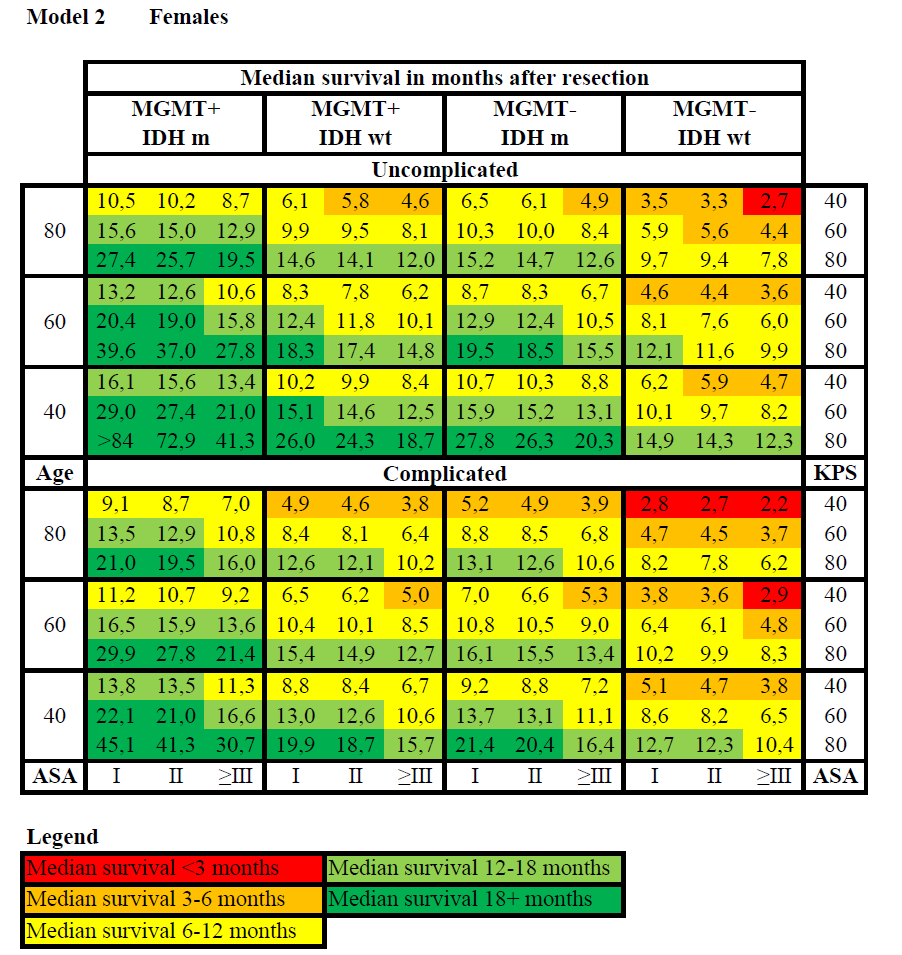

Supplement: Supplementary file 1 — Supplementary file1 (DOCX 1304 KB) [file 11060_2025_5080_MOESM1_ESM.docx]
